# Impact of tissue type and content of neoplastic cells of samples on the quality of epidermal growth factor receptor mutation analysis among patients with lung adenocarcinoma

**DOI:** 10.3892/mmr.2015.3347

**Published:** 2015-02-12

**Authors:** PANAGIOTIS PALIOGIANNIS, FEDERICO ATTENE, ANTONIO COSSU, EFISIO DEFRAIA, GIUSEPPE PORCU, ANNAMARIA CARTA, MARIA IGNAZIA SOTGIU, ANTONIO PAZZOLA, LORENZO CORDERO, FRANCESCA CAPELLI, GIOVANNI MARIA FADDA, SALVATORE ORTU, GIOVANNI SOTGIU, GRAZIA PALOMBA, MARIA CRISTINA SINI, GIUSEPPE PALMIERI, MARIA COLOMBINO

**Affiliations:** 1 Department of Surgical, Microsurgical and Medical Sciences, University of Sassari, Sassari 07100, Italy; 2 Pathology and Oncology Unit, Businco Oncological Hospital, Cagliari 09121, Italy; 3 Medical Oncology Unit, Local Health Unit (Azienda Sanitaria Locale), Sassari 07100, Italy; 4 Clinical Pulmonology and Tisiology Unit, Hospital-University (Azienda Ospedaliero Universitaria), Sassari 07100, Italy; 5 Medical Oncology Unit, ‘Zonchello’ Hospital, Nuoro 08100, Italy; 6 Medical Oncology Unit, ‘San Giovanni di Dio’ Hospital, Olbia 07026, Italy; 7 Epidemiology and Medical Statistics Unit, Department of Biomedical Sciences, University of Sassari, Sassari 07100, Italy; 8 Unit of Cancer Genetics, Institute of Biomolecular Chemistry, National Research Council (Consiglio Nazionale delle Ricerche), Sassari 07100, Italy

**Keywords:** epidermal growth factor receptor, lung adenocarcinoma, mutation analysis, specimen, tyrosine kinase inhibitors

## Abstract

Assessment of the epidermal growth factor receptor (*EGFR*) mutational status has become crucial in recent years in the molecular classification of patients with lung cancer. The impact of the type and quantity of malignant cells of the neoplastic specimen on the quality of mutation analysis remains to be elucidated, and only empirical and sporadic data are available. The aim of the present study was to investigate the impact of tissue type and content of neoplastic cells in the specimen on the quality of *EGFR* mutation analysis among patients with lung adenocarcinoma. A total of 515 patients with histologically-confirmed disease were included in the present study. Formalin-fixed paraffin embedded tissue samples were used for the mutation analysis and the content of the neoplastic cells was evaluated using light microscopy. Genomic DNA was isolated using a standard protocol. The coding sequences and splice junctions of exons 18, 19 and 21 in the *EGFR* gene were then screened for mutations by direct automated sequencing. The mean age of the patients examined was 64.9 years and 357 (69.3%) were male. A total of 429 tissue samples (83.3%) were obtained by biopsy and the remaining samples were obtained by surgery. A total of 456 samples (88.5%) were observed from primary lung adenocarcinomas, while 59 (11.5%) were from metastatic lesions. *EGFR* mutations occurred in 59 cases (11.5%); exon 18 mutations were detected in one case (1.7%), whereas exon 19 and 21 mutations were detected in 30 (51%) and 28 (47.3%) cases, respectively. *EGFR* mutations were more frequent in females and patients that had never smoked. The distribution of the mutations among primary and metastatic tissues exhibited no significant differences in the proportions of *EGFR* mutations detected. However, a statistically significant difference in the number of mutations detected was found between samples with at least 50% of neoplastic cells (450 cases-57 mutations; 12.7%) and those with <50% of neoplastic cells (65 cases-2 mutations; 3.1%).

## Introduction

Lung cancer is the malignant neoplasm with the highest incidence and is the primary cause of cancer-related mortality worldwide; with >1,800,000 novel cases and ~1,600,000 fatalities estimated in 2012 (1). Incidence rates in the general population are closely associated with the incidence of tobacco smoking, as the majority of lung cancer cases are linked to this single risk factor (2). As a consequence of the multiple campaigns adopted in previous decades against smoking in the majority of Western countries, a decrease in the incidence of lung cancer was registered in males from ~78 novel cases per 100,000 inhabitants per year between 1992–1998 to ~67/100,000 between 2005–2010 (3). However, incidence rates continuously increase in developing countries and in females, due to progressively increasing rates of smoking (3). The world standardised incidence rates of lung cancer were augmented by 22% among females and decreased by 3% among males in the period between 1985 and 2002 (4). Considering the current smoking trends, it is calculated that by 2030, lung cancer may affect females and males equally (5).

Despite recent developments in the diagnosis, clinical management and medical and surgical therapy of lung cancer, mortality rates remain high. The 5-year relative survival rate for lung cancer for the period between 1995 and 2001 was 15.7%, reflecting a steady but slow improvement from 12.5% in the period between 1974 and 1976. More recent studies have estimated a 5 year survival rate of ~16% in the USA (6,7). Several factors determine such high rates of mortality in patients with lung cancer. The most important are: i) Insufficient campaigns against smoking, pollution and other risk factors for lung cancer; ii) lack of effective screening strategies; iii) subclinical evolution of early stage disease; iv) delays in the diagnosis and clinical assessment of patients with suspicious signs and symptoms; v) insufficient comprehension of the pathophysiological mechanisms of the disease; and, as a consequence, vi) lack of effective treatment strategies, particularly for patients with advanced-stage disease.

Although the pathophysiology of lung cancer remains to be elucidated, a great quantity of research has been performed, particularly in the last two decades, and certain findings were translated into clinical practice. One of the most relevant insights was the determination of the role of the deregulation of the epidermal growth factor receptor (EGFR) for patients with non-small cell lung cancer (NSCLC). It was identified that EGFR was often overexpressed and aberrantly activated in NSCLC, and several activating mutations within the kinase domain of the *EGFR* gene were detected in lung adenocarcinomas (8). As a consequence, these tumours were highly sensitive to the EGFR-tyrosine kinase inhibitors (TKIs). TKIs were subsequently adopted into clinical practice, offering an additional therapeutic option in patients with lung adenocarcinomas. However, the increased frequency of resistance to TKIs reduced the initial enthusiasm associated with the use of this therapeutic agent (8). Nevertheless, the identification of *EGFR* mutations in patients with lung cancer remains of great importance for their clinical management and prognosis.

Assessment of the *EGFR* mutational status has therefore become a crucial step in the molecular classification of patients, with regards to treatment strategy. Different techniques are currently in use for the detection of TKI-sensitizing mutations in the *EGFR* gene at the somatic level (9). Although several approaches have been demonstrated to be more sensitive in detecting such *EGFR* gene variants [predominantly, those based on quantitative polymerase chain reaction (PCR) strategies], the most frequently used method is Sanger sequencing (10). Despite its well-recognised low sensitivity, this technique is considered the gold standard for mutational analysis (11,12). The quality of the specimen available for analysis represents a variable, which profoundly affects *EGFR* mutational classification. It is postulated that genomic DNA obtained from a quality-assessed tissue sample may markedly increase the sensitivity of the assessment, particularly when the Sanger sequencing approach is used. In addition, this factor is important in patients that do not require surgery, considering the intrinsic technical difficulties of lung biopsy methods and considering the possibility for complications that may be severe in certain instances.

The aim of the present study was to investigate the impact of the quality of the tissue sample, expressed as a percentage of neoplastic cells in the specimen, as well as the type of analysed lesions, represented by primary or secondary tumours and biopsy (transcutaneous or endoscopic) or surgical specimens, on a sequencing-based mutation analysis of the kinase domain of the *EGFR* gene in patients with lung adenocarcinomas.

## Materials and methods

### Samples

A total of 515 patients with histologically-confirmed diagnosis of NSCLC and regular follow-up in Sardinia, Italy were recruited in the present study. They were consecutively collected between September 2010 and May 2013, regardless of age at diagnosis and disease characteristics. All patients were of Sardinian origin as determined by the place of birth of the patient’s parents. Clinical and pathological features for the assessment of the disease stage at diagnosis, as well as of the onset age and anatomical location of the neoplasia, were confirmed using medical records and pathology reports. Formalin-fixed paraffin embedded (FFPE) tissue samples from NSCLC patients were obtained from the archives of the pathology institutions involved in the present study [University of Sassari, Sassari, Italy; Oncologic Hospital of Cagliari, Cagliari, Italy and the Local Health Units of Olbia (Olbia, Italy) and Nuoro (Nuoro, Italy)]. Tissue samples were evaluated for the content of neoplastic cells using light microscopy. Paraffinized sections from each patient were stained with Harris hematoxylin and aqueous eosin 1% (Leica Biosystems Richmond, Inc., Richmond, IL, USA) and examined under an Olympus BX51 optical microscope (Olympus, Center Valley, PA, USA); minimal tumoral cellularity (proportion and number of tumor cells) was established in all samples.

All patients were informed of the aims of the study and prior to collection of the tissue sample, gave written informed consent. The present study was reviewed and approved by the ethical review board of the Local Health Agency of Sassari.

### Mutation analysis

All tumour tissues were collected and processed at the laboratory of the Institute of Biomolecular Chemistry (Sassari, Italy). Genomic DNA was isolated from tissue sections using a standard protocol and DNA quality was assessed for each specimen. Paraffin was removed from FFPE samples by treatment with Bio-Clear (Bio-optica, Milan, Italy) and DNA was purified using the QIAamp DNA FFPE Tissue kit (Qiagen Inc., Valencia, CA, USA).

The coding sequence and splice junctions of exons 19 and 21 (for all cases), as well as exon 18 for a large fraction of the patients (incompleteness was due to the low quantity of available tumour tissue samples) in the *EGFR* gene were screened for mutations by direct automated sequencing. Briefly, PCR was performed on 25–50 ng of isolated genomic DNA in a 9700 Thermal cycler (Life Technologies, Carlsbad, CA, USA) using 0.5 *μ*M of each specific primer, 1.5 *μ*M MgCl_2_, 0.2 *μ*M dNTPs, and 1U AmpliTaq Polymerase (GE Healthcare Life Sciences, Piscataway, NJ, USA). PCR assays were performed by 30 cycles of denaturation at 94°C, primer annealing at 56–64°C (depending on primers), and polymerase extension at 72°C. All ll PCR-amplified products were directly sequenced using an automated fluorescence-based cycle sequencer (ABI PRISM 3100; Life Technologies), as previously described (13). Primer sequences for PCR-based assays were designed and optimised in the aforementioned laboratory and they are available upon request.

### Statistical analysis

A descriptive analysis for qualitative and quantitative variables was conducted using proportions and the mean ± standard deviation (SD), respectively. An inferential analysis was performed for the clinical and demographic variables in terms of proportion of neoplastic cells in the specimen. P≤0.05 was considered to indicate a statistically significant difference. Data were analysed using the statistical software STATA 12^®^ (StataCorp LP, College Station, TX, USA).

## Results

Among the 515 cases examined, 357 (69.3%) were male and 158 (30.7%) were female. The mean age was 64.9 years (SD:10.1). A total of 382 patients (84.5%) were active tobacco smokers or had a history of smoking, while the remaining 133 patients (15.5%) had never smoked. A total of 452 specimens (87.8%) were obtained from primary lung lesions, whereas 63 (12.2%) were obtained from metastatic lesions. The anatomical distribution of metastatic lesions was as follows: Lymph nodes, 19 (30.2%); liver, 15 (23.8%); bone, 12 (19.1%); central nervous system, 7 (11.1%); pleura, 4 (6.3%); skin, 2 (3.2%); and other tissues, 4 (6.3%). A total of 429 tissue samples (83.3%) were obtained from biopsy (transcutaneous or endoscopic), while 86 samples (16.7%) were obtained from surgical specimens.

The total number of *EGFR* mutations found was 59 (11.5%). TKI-sensitizing mutations in *EGFR* exons 18, 19, and 21 accounted for 1 (1.7%), 30 (51%) and 28 (47.3%) cases, respectively. The types of *EGFR* mutations observed are listed in Table I. The age-distribution of these mutations included 4 *EGFR*-mutations (23.5%) among the 17 patients aged <45 years. Among the other age classes, *EGFR* mutations were identified in 7/37 (18.9%) and 12/98 (12.2%) of the cases aged between 45 and 50 years and between 51 and 60 years, respectively. Approximately 10% of EGFR-mutation cases were found in patients >60 years old. The global number of EGFR mutations was significantly higher in females than in males [35/158 (22.2%) vs. 24/357 (6.7%)], due to a consistently higher incidence of EGFR exon 19 mutation in females. According to the smoking status, EGFR mutations were found to be significantly more common in patients that had never smoked (52.9%) as compared with patients that had smoked or continue to smoke (5.5%). Finally, the distribution of the mutations among primary and metastatic tissues demonstrated no statistically significant differences in the proportions of EGFR mutations detected in primary lung adenocarcinomas (51/452 mutations, 11.3%) and those found in metastatic samples (8/63 mutations, 12.7%). Table II summarises the distribution of EGFR mutations according to patient characteristics.

**Figure 1:**
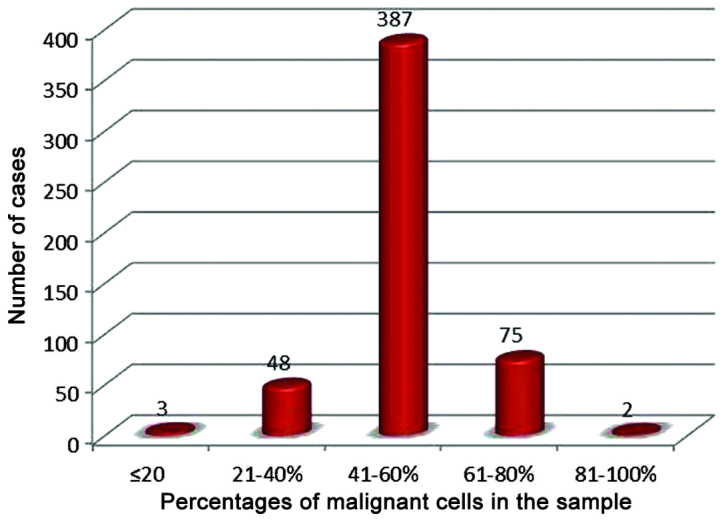
Distribution of cases on the basis of the proportions of neoplastic cells in the specimen.

The mean (SD, range) percentage of neoplastic cells in the samples employed for mutational analysis was 52.5% (9.8, 20–90). The distribution of the percentages of neoplastic cells in the specimens is shown in Fig. 1. In >75% of the cases examined, the percentage of malignant cells in the tissue sample was between 41 and 60%, while in <1% of the cases the percentage was <20% or >90%. A statistically significant difference in the number of mutations detected was found between samples with ≥50% of neoplastic cells (450 cases-57 mutations; 12.7%) and those with <50% of neoplastic cells (65 cases-2 mutations; 3.1%). Furthermore, a statistically significant difference in the proportion of malignant cells was found between samples obtained by biopsy and those obtained by surgery. The mean percentage of neoplastic cells was 50.3% (range 20–90) in biopsy specimens and 63.1% (range 40–90) in surgical samples. However, no statistical difference was inferred in *EGFR* mutation rates between tissue samples obtained by biopsy (49/429; 11.4%) or surgery (10/86; 11.6%; Table II).

## Discussion

*EGFR* mutations were found in 11.5% of Sardinian patients with lung adenocarcinoma. This percentage is similar to that reported in the literature for other Caucasian populations (14). Additionally, in the present study *EGFR* mutations were found to be significantly more frequent in females and patients that had never smoked than in males and former or active smokers. This finding has been extensively reported in numerous previous studies from different geographical areas (15–18).

The proportions of exon 18, 19 and 21 mutations were similar to those reported in the literature for other Caucasian populations (15). These mutations were frequent, other than in females and never-smokers, amongst patients aged ≤60 years at diagnosis (15.1% compared with 9.9% in patients aged >60 years at diagnosis). No statistical differences in the distribution of *EGFR* mutations were found in patients aged ≤50 years (14.1%) as compared with those aged ≥50 years (10.5%).

With regards to the origin of the tissue specimen used for mutational analysis, no statistically significant differences were identified in the percentages of *EGFR* mutations detected between samples obtained from primary tumours and those obtained from distant metastatic lesions, developed either through a lymphatic or hematogenous diffusion. In addition, no significant difference was observed in *EGFR* mutation frequencies between tissue samples obtained by biopsy and those obtained by surgery. The two findings elucidate a practical aspect for the clinical management of patients with lung cancer, as they demonstrate a clear indication that *EGFR* mutational analysis may be performed in small tissue samples obtained by biopsy methods on either primary or secondary tumour lesions, thus, avoiding the invasiveness of surgical approaches.

Several studies have investigated the effectiveness of mutation analysis performed on biopsies or fine needle aspiration samples (14,19–21). These studies, along with technological improvements in laboratory methods, confirmed the effectiveness of EGFR mutational analysis in small tumour samples. This finding was also confirmed in the present study. Malapelle *et al* (22) in a previous study compared EGFR mutation analysis in 318 histology samples with that performed on 364 cytology specimens; the authors registered 8.5% and 8.8% of total EGFR mutations in the histological and cytological samples, respectively.

To the best of our knowledge, the impact of the quantity of neoplastic cells on mutation analysis has not been previously investigated thoroughly, and only empirical and sporadic data are available. Recommendations of several scientific societies on the minimum quantity of neoplastic cells required in the specimen for an adequate mutational analysis are generally based on such data. The Italian guidelines produced recently by a collaboration of three different scientific societies (AIOM-SIAPEC-IAP) recommend that when standard mutational analysis procedures are used (direct sequencing) the sample should be composed of at least 50% neoplastic cells (23).

As has been previously mentioned, in the present data, the majority of the samples examined comprised >50% neoplastic cells. It was identified that specimens with >50% neoplastic cells correlated positively with a higher number of *EGFR* mutations detected. This finding confirms that what is important is not whether specimens are taken during biopsy or surgery or whether they are taken from primary or metastatic tumours, but the quality of its cell composition. This appears to be the main aspect, which pathologists should focus on when evaluating or preparing specimens for mutation analysis. Additionally, standardised and reproducible methods must be outlined for a precise evaluation of the percentage of malignant cells in neoplastic specimens, in order to avoid confusion due to the different methods and criteria currently in use.

## Figures and Tables

**Table I:** Sequence variations in epidermal growth factor receptor gene among the 59 mutated cases found.

No. of positive cases (%)	*EGFR* exon	DNA change	Amino acid change	Effect	Designation
1 (1.7)	18	c.2154 G>C	p.Gly719Ala	Missense	G719A
22 (37.2)	19	c.2235-2249 del15	p.del746_750	In-frame deletion	delELREA
1 (1.7)	19	c.2237_2255 del18>TT	p.del746_S752>V	In-frame deletion complex	delELREATS>V
1 (1.7)	19	c.2235_2249 del15	p.del745_750	In-frame deletion	delKELREA
1 (1.7)	19	c.2239_2251 del13; 2253_2254insA	p.del747_750	In-frame deletion complex	delLREA
1 (1.7)	19	c.2239_2248 del10; 2248_2249ins	p.del747_749	In-frame deletion complex	delLRE
1 (1.7)	19	c.2239_2253 del15	p.del747_751	In-frame deletion	delLREAT
2 (3.4)	19	c.2252C>G; 2253_2277 del24	p.T751S; del752_759	In-frame deletion complex	delSPKANKEI
1 (1.7)	19	c.2239_2240 TT>CC	p.Leu747Pro	Missense	L747P
1 (1.7)	21	c.2572 C>A	p.Leu858Met	Missense	L858M
1 (1.7)	21	c.2582 T>A	p.Leu861Gln	Missense	L861Q
26 (44.1)	21	c.2573 T>G	p.Leu858Arg	Missense	L858R

**Table II:** Distribution of epidermal growth factor receptor mutations according to patient characteristics.

Characteristic	No. of patients	No. of *EGFR* mutated cases (%)
Total analysed	515	59 (11.5)
Males/females	357/158	24/35 (6.7/22.2)
Age (years)		
<45	17	4 (23.5)
45–50	37	7 (18.9)
51–60	98	12 (12.2)
>60	363	36 (9.9)
Smoking status		
Smoker	174	6 (3.4)
Former smoker	209	15 (7.2)
Never smoked	70	37 (52.9)
Unknown	62	1 (1.6)
Tumour status		
Primary tumour	452	51 (11.3)
Metastasis		
Sample type	63	8 (12.7)
Biopsy	429	49 (11.4)
Surgical	86	10 (11.6)

## References

[1] Ferlay J, Shin HR, Bray F, Forman D, Mathers C, Parkin DM (2012). GLOBOCAN 2012 v1.2, Cancer Incidence and Mortality Worldwide: IARC CancerBase No. 10 (Internet).

[2] Alberg AJ, Brock MV, Ford JG, Samet JM, Spivack SD (2013). Epidemiology of lung cancer: Diagnosis and management of lung cancer, 3rd ed: American college of chest physicians evidence-based clinical practice guidelines. Chest.

[3] Paliogiannis P, Attene F, Cossu A (2013). Lung cancer epidemiology in North Sardinia, Italy. Multidiscip Respir Med.

[4] Parkin DM, Bray F, Ferlay J, Pisani P (2005). Global cancer statistics, 2002. CA Cancer J Clin.

[5] Jemal A, Siegel R, Xu J, Ward E (2010). Cancer statistics, 2010. CA Cancer J Clin.

[6] Alberg AJ, Ford JG, Samet JM (2007). American college of chest physicians. Epidemiology of lung cancer: ACCP evidence-based clinical practice guidelines (2nd edition). Chest.

[7] Lewis DR, Chen HS, Feurer EJ (2010). SEER Cancer statistics review, 1975–2008.

[8] Yamamoto H, Toyooka S, Mitsudomi T (2009). Impact of EGFR mutation analysis in non-small cell lung cancer. Lung Cancer.

[9] Thunnissen E, Kerr KM, Herth FJ (2012). The challenge of NSCLC diagnosis and predictive analysis on small samples. Practical approach of a working group. Lung Cancer.

[10] Ellison G, Zhu G, Moulis A, Dearden S, Speake G, McCormack R (2013). EGFR mutation testing in lung cancer: a review of available methods and their use for analysis of tumour tissue and cytology samples. J Clin Pathol.

[11] Tsiatis AC, Norris-Kirby A, Rich RG, Hafez MJ, Gocke CD, Eshleman JR, Murphy KM (2010). Comparison of Sanger sequencing, pyrosequencing, and melting curve analysis for the detection of KRAS mutations: diagnostic and clinical implications. J Mol Diagn.

[12] Anderson S, Bloom KJ, Vallera DU (2012). Multisite analytic performance studies of a real-time polymerase chain reaction assay for the detection of BRAF V600E mutations in formalin-fixed paraffin-embedded tissue specimens of malignant melanoma. Arch Pathol Lab Med.

[13] Palomba G, Colombino M, Contu A (2012). Prevalence of KRAS, BRAF and PIK3CA somatic mutations in patients with colorectal carcinoma may vary in the same population: clues from Sardinia. J Transl Med.

[14] Nana-Sinkam SP, Powell CA (2013). Molecular biology of lung cancer: Diagnosis and management of lung cancer, 3rd ed: American college of chest physicians evidence-based clinical practice guidelines. Chest.

[15] Pao W, Miller V, Zakowski M (2004). EGF receptor gene mutations are common in lung cancers from ‘never smokers’ and are associated with sensitivity of tumors to gefitinib and erlotinib. Proc Natl Acad Sci USA.

[16] Shigematsu H, Lin L, Takahashi T (2005). Clinical and biological features associated with epidermal growth factor receptor gene mutations in lung cancers. J Natl Cancer Inst.

[17] Fukuoka M, Yano S, Giaccone G (2003). Multi-institutional randomized phase II trial of gefitinib for previously treated patients with advanced non-small-cell lung cancer (The IDEAL 1 Trial) [corrected]. J Clin Oncol.

[18] Miller VA, Kris MG, Shah N (2004). Bronchioloalveolar pathologic subtype and smoking history predict sensitivity to gefitinib in advanced non-small-cell lung cancer. J Clin Oncol.

[19] Hantson I, Dooms C, Verbeken E (2014). Performance of standard procedures in detection of EGFR mutations in daily practice in advanced NSCLC patients selected according to the ESMO guideline: a large Caucasian cohort study. Transl Respir Med.

[20] Vigliar E, Malapelle U, Bellevicine C (2014). Outsourcing cytological samples to a referral laboratory for EGFR testing in non-small cell lung cancer: does theory meet practice?. Cytopathology.

[21] Lozano MD, Labiano T, Echeveste J (2014). Assessment of EGFR and KRAS mutation status from FNAs and core-needle biopsies of nonsmall cell lung cancer. Cancer Cytopathol.

[22] Malapelle U, Bellevicine C, De Luca C (2013). EGFR mutations detected on cytology samples by a centralized laboratory reliably predict response to gefitinib in non-small cell lung carcinoma patients. Cancer Cytopathol.

[23] Marchetti A, Normanno N, AIOM-SIAPEC-IAP (2010). Recommendations for mutational analysis of EGFR in lung carcinoma. Pathologica.

